# Low Dose CT Image Reconstruction Based on Structure Tensor Total Variation Using Accelerated Fast Iterative Shrinkage Thresholding Algorithm

**DOI:** 10.3390/s20061647

**Published:** 2020-03-16

**Authors:** Junfeng Wu, Xiaofeng Wang, Xuanqin Mou, Yang Chen, Shuguang Liu

**Affiliations:** 1 Department of Applied Mathematics, Xi’an University of Technology, Xi’an 710048, China; xfwang@xaut.edu.cn; 2 The Key Laboratory of Computer Network and Information Integration, Southeast University and Ministry of Education, Nanjing 210096, China; chenyang.list@seu.edu.cn; 3 The Institute of Image processing and Pattern recognition, Xi’an Jiaotong University, Xi’an 710049, China; xqmou@mail.xjtu.edu.cn; 4 Equipment Management and Unmanned Aerial Vehicle Engineering College, Air Force Engineering University, Xi’an 710051, China; dawny418@126.com

**Keywords:** low dose CT, structure tensor total variation, accelerated fast iterative shrinkage thresholding

## Abstract

Low dose computed tomography (CT) has drawn much attention in the medical imaging field because of its ability to reduce the radiation dose. Recently, statistical iterative reconstruction (SIR) with total variation (TV) penalty has been developed to low dose CT image reconstruction. Nevertheless, the TV penalty has the drawback of creating blocky effects in the reconstructed images. To overcome the limitations of TV, in this paper we firstly introduce the structure tensor total variation (STV_1_) penalty into SIR framework for low dose CT image reconstruction. Then, an accelerated fast iterative shrinkage thresholding algorithm (AFISTA) is developed to minimize the objective function. The proposed AFISTA reconstruction algorithm was evaluated using numerical simulated low dose projection based on two CT images and realistic low dose projection data of a sheep lung CT perfusion. The experimental results demonstrated that our proposed STV_1_-based algorithm outperform FBP and TV-based algorithm in terms of removing noise and restraining blocky effects.

## 1. Introduction

The X-ray computer tomography (CT) has been extensively utilized in industry nondestructive testing and medical diagnosis. However, repeated use of conventional CT could significantly increase the chance of developing cancer and other diseases due to the high radiation exposure [1,2,3]. Hence, low dose CT which was firstly proposed by Naidich [4], has become one of the research hot spots in the CT field. Lowering the tube current values (milliampere (mA) or milliampere second (mAs)) or the voltage values (kilovolt (kV)) is the most straightforward way to reduce the radiation dose because it does not need to change the scanning structure of existing CT equipment. Nonetheless, this method will result in insufficient number of x-ray photons detected at the detector and hence, upgrade the quantum noise level on the sinogram. In this situation, for most current commercial CT scanners, the often used Feldkamp-Davis-Kress algorithm (or its variants) will lead to severe image quality degradation due to noisy projection. Hence, it is highly desirable to develop a new method to reconstruct the high-quality image for LDCT imaging.

Due to its advantages in effective physical noise modeling and possibilities of incorporating priors of the reconstructed image, the statistical iterative reconstruction (SIR) method [5,6] had been shown to be superior in removing image noise and streak artifacts. Recently, inspired by compressed sensing (CS) theory [7], in 2008, Sidky et al. [8] first introduced the total variation (TV) into a low dose CT reconstruction field and proposed an adaptive-steepest-descent-projection onto convex sets (ASD-POCS) algorithm. Subsequently, Tang et al. [9] introduced the TV regularization term into SIR framework for low dose CT reconstruction and further developed a Gauss-Siedel iterative algorithm to minimize the objective function. Choi et al. [10] investigated a primal-dual first-order method to minimize the TV-based SIR framework for CBCT image reconstruction with sparse and noisy low-dose projection data. However, the TV regularization term has the drawback of creating staircase artifacts, particularly at low dose levels. To further eliminate the block effects, Xu et al. [11] proposed a dictionary learning-based approach for low dose CT reconstruction, in which the sparse constraint in terms of a redundant dictionary was incorporated into an objective function in a SIR reconstruction framework, and further proposed an alternating minimization reconstruction algorithm. Sun et al. [12] proposed a Hessian penalty and developed an effective algorithm to minimize the objective function using a maximization-minimization (MM) approach. Zhang et al. [13] described two iterative reconstruction algorithms for low dose CT image reconstruction based on a Gamma regularization. Shangguan et al. [14] introduced a modified Markov random field regularization term into the SIR framework and utilized a modified alternation iterative algorithm to optimize the cost function. Very recently, Shi et al. [15] combined the weighted TV and Hessian penalties for low dose CT reconstruction in a structure-adaptive way, in which a MM approach was designed to optimize the objection function. Xu et al. [16] introduced the TV and wavelet-based L_1_ penalties into SIR framework and solved the objective function using accelerated variants of the fast iterative soft-thresholding algorithm. Zhang et al. [17] introduced an adaptive fractional order TV penalty and used a separable paraboloid surrogate (SPS) method to minimize the objection function. Liu et al. [18] discussed and compared the behaviors of several convex Hessian Schatten penalties with orders 1,2 and ∞ for low dose CT reconstruction. At the same time, K. Kim et al. [19] proposed low dose CT reconstruction using spatially encoded nonlocal penalty, in which an ordered subset SQS method for log-likelihood is developed and the patch-based similarity constraint with a spatially variant factor is developed to reduce the noise effectively and preserve features simultaneously. Very recently, Cai et al. [20] investigated block-marching sparsity regularization and developed a practical reconstruction algorithm using hard thresholding and projection onto convex set methods for low dose CT reconstruction.

In 2015, S. Lefkimmiatis et al. [21] proposed a novel structure tensor total variation (STV) penalty for image denoising and image deblurring. In contrast to TV penalty, STV penalized the image variation at every point of the domain by taking into account the information in a local neighborhood. Hence, it supplies a richer and more robust measure of image variation which translates to improve reconstruction performance. Inspired by this work, Zeng et al. [22] firstly introduced STV penalty into medical imaging field for multi-energy CT reconstruction. Later, Zeng et al. [23] developed a robust perfusion deconvolution approach via STV regularization for estimating an accurate residue function in cerebral perfusion CT with low mAs data acquisition. In 2018, Gong et al. [24] developed a SIR approach by incorporating a precontrast normal-dose CT scan of robust dynamic myocardial perfusion CT. Inspired by the above research, in this paper we introduce the STV penalty into the SIR framework for low dose CT reconstruction, and then develop an accelerated fast iterative shrinkage thresholding algorithm (AFISTA) to minimize the associated objective function. At last, the resulting method is evaluated using simulated low dose projection data and real CT projection data

The remainder of this paper is organized as follows. Section 2 describes the SIR, SIR framework with STV_1_ penalty, and AFISTA for the proposed reconstruction model. Section 3 illustrates the performance of the proposed reconstruction approach via two numerical simulated experiments and realistic CT projection. Finally, we will discuss relevant issues and conclude this paper in Section 4.

## 2. Materials and Methods

### 2.1. SIR

The SIR algorithm [5] for reconstructing a CT image u can be expressed as the following minimization problem:

(1) $$u^{*}=\underset{u}{\arg\min}\left\{\Phi (u)≜\frac{1}{2}{\left\|Au-p\right\|}_{W}^{2}+\beta R(u)\right\}$$

where $u={\left(u_{1},u_{2},\ldots ,u_{N}\right)}^{T}$ is the image vector representing the attenuation coefficients of the imaged object, $p={\left(p_{1},p_{2},\ldots ,p_{m}\right)}^{T}$ is the vector representing the raw detector measurements, A is the m×N system matrix that models the imaging system, the diagonal matrix W provides statistical weighting that accounts for the ray-dependent variance of the noise, R(u) is the function of the image u which is called regularization term, β is the regularization parameter that balances the data-fitting term and the regularization term, and Φ(u) is the objective function.

### 2.2. SIR with STV_1_ Penalty for Low Dose CT Reconstruction

Discrete STV_1_ penalty [21] can be expressed as:

(2) $${\mathrm{STV}}_{1}(u)=\sum_{n=1}^{N}{\left\|{\left[J_{K}u\right]}_{n}\right\|}_{S_{1}}$$

where u denotes an image, N denotes the number of pixels in the image u, ${\left\|\cdot \right\|}_{S_{1}}$ denotes the Schatten nuclear norm, corresponding to the $S_{1}$ norm of the singular values, $J_{K}$ is the patch-based Jacobian operator of the image u, which is defined as the linear mapping $J_{K}:R^{N}\mapsto 𝒳$, where $𝒳≜R^{N\times L\times 2}$, K is a non-negative, rotationally symmetric convolution kernel, ${\left[J_{K}u\right]}_{n}$ denotes a matrix with the patch-based Jacobian applied on the *nth* pixel of the u, which is defined as

(3) $${\left[J_{K}u\right]}_{n}={\left({\left[T_{s_{1},w}\circ \nabla u\right]}_{n},\ldots ,{\left[T_{s_{L},w}\circ \nabla u\right]}_{n}\right)}^{T}$$

where ${\left(\cdot \right)}^{T}$ is the transpose operator, ∇ denotes the discrete gradient operator, ∘ denotes the composition of operator. The shift vectors $s_{l}(l=1,\ldots ,L)$ are the elements in the neighborhood of the *nth* pixel, where $L={\left(2L_{k}+1\right)}^{2}$, $L_{k}$ is the radius of the convolution kernel, $T_{S_{l},w}$ is a weighted translation operator, which is defined as:

(4) $${\left[T_{{}_{S_{l},w}}\circ \nabla u\right]}_{n}=w(s_{l})\nabla u[x_{n}-s_{l}]$$

where $x_{n}$ represents the coordinates of image u and $w(s)=\sqrt{K[s]}$ denotes the window function of the convolution kernel [21]. In this work, we chose Gaussian kernel to design the structure tensor.

To overcome the block effects of TV penalty and inspired by the successful application of STV_1_ penalty in image processing field [21], a new SIR-STV_1_ framework for low dose CT reconstruction is proposed as follows:

(5) $$u^{*}=\arg\underset{u}{\min}\left\{\Phi (u)≜\frac{1}{2}{\left\|Au-p\right\|}_{W}^{2}+\beta {\mathrm{STV}}_{1}(u)\right\}$$

### 2.3. AFISTA Algorithm for Solving SIR-STV_1_

#### 2.3.1. General FISTA for Solving SIR-STV_1_

FISTA has been widely used for image denoising and deblurring in image processing field due to its ability to minimize a cost function that is specified by the sum of a smooth and convex data fidelity term and a convex but possibly nonsmooth penalty [25]. Since the STV_1_ penalty is not differentiable everywhere, the traditional gradient-based algorithm cannot be directly applied to minimize the cost function (5). Therefore, in this paper we propose to adapt the FISTA algorithm to solve problem (5).

Let

(6) $$F(u)=\frac{1}{2}{\left\|Au-p\right\|}_{w}^{2}$$

the FISTA algorithm decoupled the minimization problem (5) into two steps. The first step is to minimize the data fitting term $F(y^{\left(i\right)})$ using a gradient descent method, which can be expressed as:

(7) $$v^{\left(i\right)}=y^{\left(i\right)}-\frac{1}{Q}\nabla F(y^{\left(i\right)})=y^{\left(i\right)}-\frac{1}{Q}A^{T}W(Ay^{\left(i\right)}-p)$$

where *i* is the iteration number, *Q* is the Lipschitz constant of ∇F(u). The second step is STV_1_-based denoising problem, which can be represented as:

(8) $$u^{\left(i\right)}=\underset{u}{\arg\min}\left\{\frac{1}{2}{\left\|u-v^{\left(i\right)}\right\|}_{2}^{2}+\frac{\beta }{Q}{\mathrm{STV}}_{1}(u)\right\}$$

Let λ=β/Q, Equation (8) can be solved efficiently by a progressive proximal map algorithm [21]. In all, the detailed workflow of the general FISTA is listed in Algorithm 1.
**Algorithm 1** Workflow of the general fast iterative shrinkage thresholding algorithm (FISTA).**Input:** system matrix A, projection data p, *Q* is the Lipschitz constant of ∇F(u)**Initial Step:**$y^{\left(1\right)}=u^{\left(0\right)}=0$, $t_{1}=1$, maximum iteration number *I*, regularization parameter λ, convolution kernel K**for***i* = 1,2,...,*I*  Update intermediate image $v^{\left(i\right)}$ with (7)  Update image $u^{\left(i\right)}$ with (8)  $t_{i+1}=\frac{1}{2}(1+\sqrt{1+4t_{i}^{2}})$
  $y^{\left(i+1\right)}=u^{\left(i\right)}+((t_{i}-1)/t_{i+1})/(u^{(i}{}^{)}-u^{\left(i-1\right)})$
**end for****output:**$u^{\left(I\right)}$

#### 2.3.2. AFISTA for Solving SIR-STV_1_

Although the general FISTA algorithm can be applicable to solve problem (5), it poses challenges for optimization. Lipschitz constant of the gradient data-fitting term is very large for low dose CT reconstruction [6,26,27], which results in small gradient steps leading to slow convergence. To solve this problem, we proposed to use the separable quadratic surrogate (SQS) method [6,28], that replaces the data fitting term $F(y^{\left(i\right)})$ by a surrogate function $\phi (v;y^{\left(i\right)})$ at *i*th iteration.

(9) $$\phi (v;y^{\left(i\right)})≜F(y^{\left(i\right)})+\nabla F(y^{\left(i\right)})(v-y^{\left(i\right)})+\frac{D}{2}{\left(v-y^{\left(i\right)}\right)}^{2}$$

where $D≜diag\{[A^{T}WA]1\}$ is a diagonal Hessian (second order derivatives) matrix. $\phi (v;y^{\left(i\right)})$ can be easily minimized by zeroing the first derivative, this leads to the following updating algorithm:

(10) $$v^{\left(i+1\right)}=y^{\left(i\right)}-D^{-1}\nabla F(y^{\left(i\right)})=y^{\left(i\right)}-D^{-1}A^{T}W(Ay^{\left(i\right)}-p)$$

To further accelerate updating Equation (10), we adopt order subset (OS) methods [29], by grouping the projection views into *M* subsets evenly and using only the subset of measured data to approximate the exact gradient of the cost function.

Furthermore, to find the solution of Equation (8), we first introduce a dual norm of ${\mathrm{STV}}_{1}(u)$ and write it as follows [21]:

(11) $$\sum_{n=1}^{N}{\left\|{\left[J_{K}u\right]}_{n}\right\|}_{S_{1}}=\underset{\Omega \in B_{\infty ,\infty }}{\max}{\langle \Omega ,J_{K}u\rangle }_{𝒳}$$

where $𝒳≜{\mathbb{R}}^{N\times L\times 2}$ is the target space of $J_{K}$, Ω denotes the variable in the target space 𝒳, $B_{\infty ,\infty }$ denotes the $l_{\infty }-S_{\infty }$ unit-norm ball, which can be expressed as:

(12) $$B_{\infty ,\infty }=\{\Omega \in 𝒳:\|\Omega_{n}\|{}_{S_{n}}\leq 1,\forall n=1,\ldots ,N\}$$

Then, the proximal map of STV_1_ can be rewritten as follows:

(13) $$u^{*}=\underset{u\in C}{\arg\min}\frac{1}{2}{\left\|u-v\right\|}_{2}^{2}+\lambda \underset{\Omega \in B_{\infty ,\infty }}{\max}{\langle J_{K}^{*}\Omega ,u\rangle }_{2}$$

where convex set $C={\mathbb{R}}^{N}$, ${\langle J_{K}^{*}\Omega ,u\rangle }_{2}={\langle \Omega ,J_{K}u\rangle }_{𝒳}$ and $J_{K}^{*}$ denotes the adjoint of the patch-based Jacobian operator. This formulation naturally leads us to the following minmax problem:

(14) $$\underset{u\in C}{\min}\underset{\Omega \in B_{\infty ,\infty }}{\max}ℒ(u,\Omega )=\frac{1}{2}{\left\|u-v\right\|}_{2}^{2}+\lambda {\langle J_{K}^{*}\Omega ,u\rangle }_{2}$$

Since ℒ is a strictly convex w.r.t. u and concave w.r.t. Ω, a saddle point of ℒ can be obtained. Therefore, the order of the minimum and the maximum in Equation (13) does not affect the solution. This means that there exists a common saddle point $\left(u^{*},\Omega^{*}\right)$ when the minimum and the maximum are interchanged, i.e.,

(15) $$\underset{u\in C}{\min}\underset{\Omega \in B_{\infty ,\infty }}{\max}ℒ(u,\Omega )=ℒ(u^{*},\Omega^{*})=\underset{\Omega \in B_{\infty ,\infty }}{\max}\underset{u\in C}{\min}ℒ(u,\Omega )$$

Based on Equation (15), two optimization problems, the primal and the dual one can be defined. This can be accomplished by identifying the primal and dual objective functions, respectively, with the following minmax problem [21]:

(16) $$p(u)=\underset{\Omega \in B_{\infty ,\infty }}{\max}ℒ(u,\Omega )=\frac{1}{2}{\left\|u-v\right\|}_{2}^{2}+\lambda \sum_{n=1}^{N}{\left\|{\left[J_{K}u\right]}_{n}\right\|}_{S_{1}}$$

(17) $$d(\Omega )=\underset{u\in C}{\min}ℒ(u,\Omega )=\frac{1}{2}{\left\|z-\prod_{C}\left(z\right)\right\|}_{2}^{2}+\frac{1}{2}\left({\left\|v\right\|}_{2}^{2}-{\left\|z\right\|}_{2}^{2}\right)$$

where $z=v-\lambda J_{K}^{*}\Omega$ and $\prod_{C}$ is the orthogonal projection operator on the convex set C. According to the conclusion in Ref. 21, the minimizer $u^{*}$ of the primal objective function can be obtained from the maximizer $\Omega^{*}$ of the dual objective function. It can be expressed as follows:

(18) $$u^{*}=\prod_{C}(v-\lambda J_{K}^{*}\Omega^{*})$$

where $\Omega^{*}$ can be derived as the optimization of the corresponding dual objective function in Equation (17). For minimizing the dual objective function in Equation (17), the progressive proximal map algorithm [21] can be used and listed as follows (Algorithm 2):
**Algorithm 2** Workflow of the progressive proximal map algorithm**Input:**v, λ, $\prod_{C}$**Initial Step:**$\Psi^{\left(1\right)}=\Omega^{\left(0\right)}=0$, $t_{1}=1$**for***i* = 1,2,...,*I*  $\Omega^{\left(i\right)}=\prod_{B\infty ,\infty }(\Psi^{\left(i\right)}+\frac{1}{8\sqrt{2}}J_{K}\prod_{C}(v-\lambda J_{{}_{K}}^{*}\Psi^{\left(i\right)}))$
  $t_{i+1}=\frac{1}{2}(1+\sqrt{1+4t_{i}^{2}})$
  $\Psi^{\left(i+1\right)}=\Omega^{\left(i\right)}+((t_{i}-1)/t_{i+1})/(\Omega^{\left(i\right)}-\Omega^{\left(i-1\right)})$**end for****Output**$u=\prod_{C}(v-\lambda J_{K}^{*}\Omega^{\left(I-1\right)})$

In all, the proposed AFISTA can be listed as follows (Algorithm 3):
**Algorithm 3** Workflow of the proposed accelerated (A)FISTA**Input:** System matrix A, projection data p, v, λ, $\prod_{C}$
**Initial Step:**$y^{\left(1\right)}=u^{\left(0\right)}=0$, $t_{1}=1$, maximum iteration number *I*, regularization parameter λ, convolution kernel *K,* the number of order subset *M***for***i* = 1,2...*I* **for**
*m* = 1,...*M*  k=(i−1)×I+m
  Update intermediate image using Equation (10):  $v^{\left(k\right)}=y^{\left(k\right)}-D^{-1}MA_{m}^{T}W_{m}(A_{m}y^{\left(k\right)}-p_{m})$
  $A_{m}$, $W_{m}$, $p_{m}$ are submatrices of A, W, p corresponding to the mth subset  Update image $u^{\left(k\right)}$ with progressive proximal map algorithm using Algorithm 2  $t_{k+1}=\frac{1}{2}(1+\sqrt{1+4t_{k}^{2}})$  $y^{\left(k+1\right)}=u^{\left(k\right)}+((t_{k}-1)/t_{k+1})/(u^{(k}{}^{)}-u^{\left(k-1\right)})$
 **end for****end for****Output**$u^{\left(I\times M\right)}$

## 3. Experimental Results

In this subsection, a series of numerical simulation experiments were designed to evaluate the performance of the proposed method in CT image reconstruction from a low dose situation. In addition, low dose and under sampled raw projections of a sheep lung perfusion were acquired to validate the feasibility of our proposed method in practical applications. Meanwhile, the TV-based SIR (denoted by SIR-TV) and filtered backprojection(FBP) methods were presented for comparison.

### 3.1. Brain Image Numerical Simulation 

**Figure 1:**
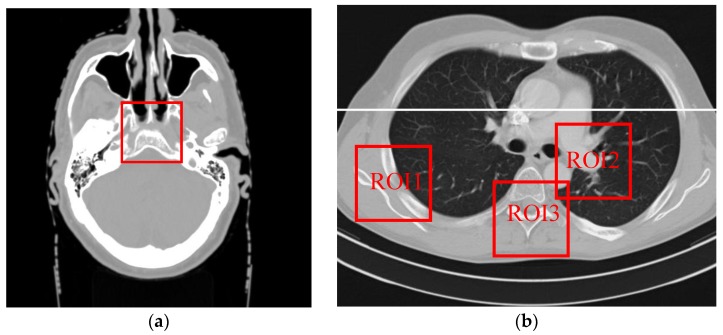
Two phantoms (**a**) brain image phantom; (**b**) Thorax image phantom. The display window is [−1000, 667] Hounsfield Unit(HU).

A human brain CT image, downloaded from website [30], was used to validate the performance of the proposed algorithm. This image was a 2 mm thick corrected form of a female patient. During scanning, the X-ray tube voltage and tube current was 120 KVp and 200 mAs, respectively, a monochromatic spectrum was adopted, scattered photons were not considered, and the equi-angularly detector was used. The image is shown in Figure 1a. The spatial dimension is 512 × 512 pixels and the size of each pixel is 0.4883 × 0.4883 mm. 

In this simulation, we adopt the fan-beam geometry with an equi-angular detector to simulate projection. The distance from the X-ray source to the detector is 1140 mm and the distance from the rotation center to the curved detector is 570 mm. Uniformly collected in [0, 2π] were 1160 projections. For each projection, 672 detector elements spanned a field-of-view (FOV) of 25 cm in radius. To obtain noisy projection, we firstly compute noiseless projection using siddons’s ray-driven algorithm. Then, Poisson noise assuming 5 × 10^3^, 1 × 10^4^, and 5 × 10^4^ photons per detector element was superimposed on the obtained noiseless projection to simulated three different noise levels, respectively. 

#### 3.1.1. Convergence Analysis

**Figure 2:**
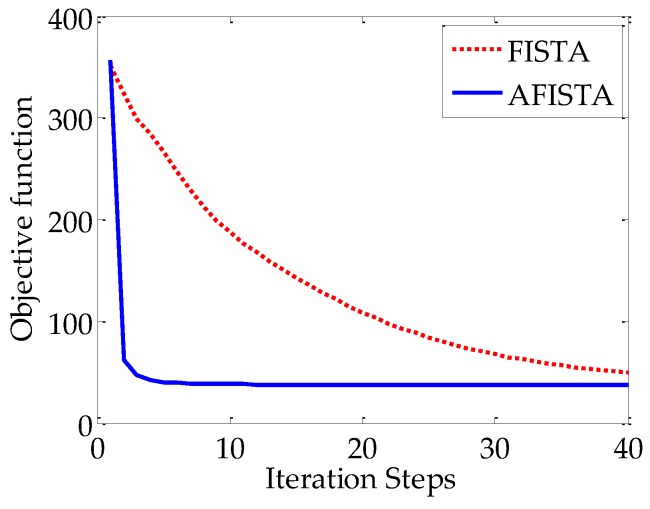
The value of objective function in terms of iteration steps of the proposed AFISTA methods and FISTA for the brain image phantom with 5 × 10^4^ incident photon numbers.

To examine the convergence of our proposed algorithm, Figure 2 shows the value of objective function in terms of iteration steps for the brain image numerical simulation with 5 × 10^4^ incident photon numbers. The curve indicates that our proposed algorithm can converge to a steady solution after 20 iterations. Furthermore, it can be seen that the proposed AFISTA method converges much faster than FISTA.

#### 3.1.2. Visual Quality Comparison

**Figure 3:**
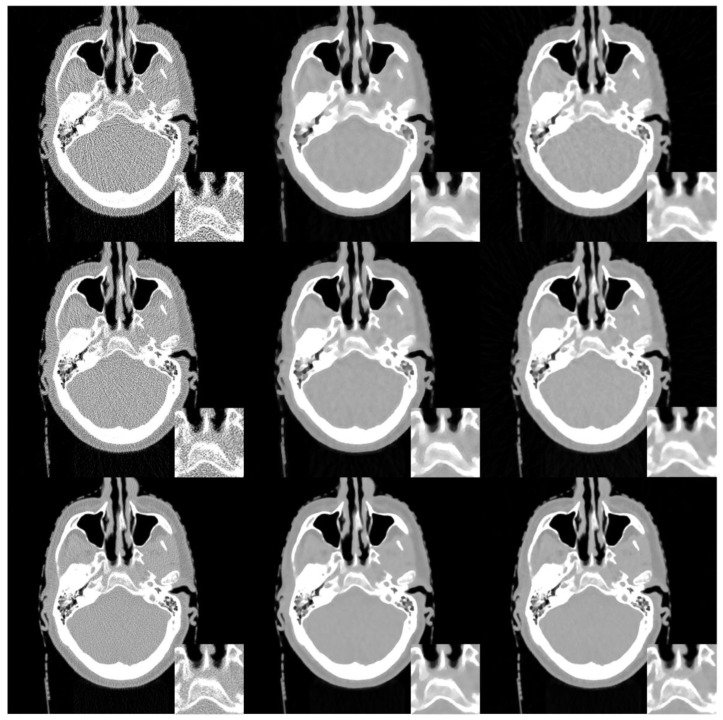
Images reconstructed by the FBP (left column), SIR-TV (middle column), and structure tensor total variation SIR-STV_1_ (right column) methods from noisy projections with incident photon numbers 5 × 10^3^ (the first row), 1 × 10^4^ (the second row), and 5 × 10^4^ (the third row). The display window is [−1000, 667] HU.

Figure 3 shows the reconstructed brain images and zoomed regions of interest(ROI) (indicated by the red square in Figure 1a) reconstructed from low dose projections. Images in the left, middle, and right column are reconstructed by the FBP, SIR-TV, and SIR-STV_1_ method, respectively. Images in the first, second, and third row are reconstructed from noisy projections with incident photon numbers 5 × 10^3^, 1 × 10^4^, and 5 × 10^4^, respectively. In the SIR-STV_1_ method, parameters σ and $L_{K}$ are selected according to the method described in Ref. [21], i.e.,σ=0.5, $L_{K}=3$, the number of subset were set to 40. For the reconstruction from noisy projections with incident photon numbers 5 × 10^3^, 1 × 10^4^, and 5 × 10^4^, the parameter λ were set as 6 × 10^−5^, 2 × 10^−5^, and 9 × 10^−6^, respectively. It can be seen that the FBP results contains serious noise, and became worse and worse when the number of photon decreased from 5 × 10^4^ to 5 × 10^3^. The SIR-TV method removes noise but the reconstructed image suffers from over smoothing effect. The reconstructed results using the SIR-STV_1_ perform better in restraining the blocky effect and removing image noise than other methods, and are almost visually the same as the original image. 

#### 3.1.3. Quantitative Comparison

To quantify the reconstruction accuracy of the low dose reconstruction algorithm, signal-to-noise ratio (PSNR) [31], relative reconstruction error (RRE), and structure similarity (SSIM) [32] indexes are employed in this subsection.

(19) $$\mathrm{PSNR}=10\log_{10}\frac{{\left(\max(\mu_{n})\right)}^{2}}{\sum_{n}{\left(\mu_{n}-\mu_{n}^{*}\right)}^{2}/N}$$

(20) $$\mathrm{RRE}=\frac{{\left\|\mu -\mu^{*}\right\|}_{2}^{2}}{{\left\|\mu \right\|}_{2}^{2}}$$

(21) $$\mathrm{SSIM}=\frac{\left(2\bar{\mu }{\bar{\mu }}^{*}+c_{1})(2\sigma_{\mu \mu^{*}}+c_{2}\right)}{\left({\bar{\mu }}^{2}-{\bar{\mu }}^{*}{}^{2}+c_{1})(\sigma_{\mu }^{2}+\sigma_{\mu^{*}}^{2}+c_{2}\right)}$$

where $\mu_{n}$ is the reconstructed value, $\mu_{n}^{*}$ is the golden reference value,J is the number of the pixels in the reconstructed image, $\bar{\mu }$ and ${\bar{\mu }}^{*}$ are the mean value of μ and $\mu_{}^{*}$, $\sigma_{\mu }^{}$ and $\sigma_{\mu^{*}}^{}$ are the variation of μ and $\mu_{}^{*}$, $\sigma_{\mu \mu^{*}}$ is the covariance of μ and $\mu_{}^{*}$, $c_{1}$ and $c_{1}$ are the constants that we choose according to Reference [32]. Table 1 gives the PSNR, RRE, and SSIM values of the reconstructed whole brain images in Figure 3. It can be seen that the SIR-STV_1_ method has the best performance in all evaluation metrics in all noise levels.

### 3.2. Thorax Image Numerical Simulation 

To further validate the performance of the SIR-STV_1_ method, a human Thorax image is downloaded from website [33], which is shown in Figure 1b. The spatial dimensional of the image is 512 × 512 pixels, the real size of each pixel and the imaging geometry are exactly the same as that used in the brain numerical simulation. The Poisson noise assuming 5 × 10^4^ photons per detector element was added to simulate noisy projection. 

#### 3.2.1. Visual Quality Comparison

**Figure 4:**
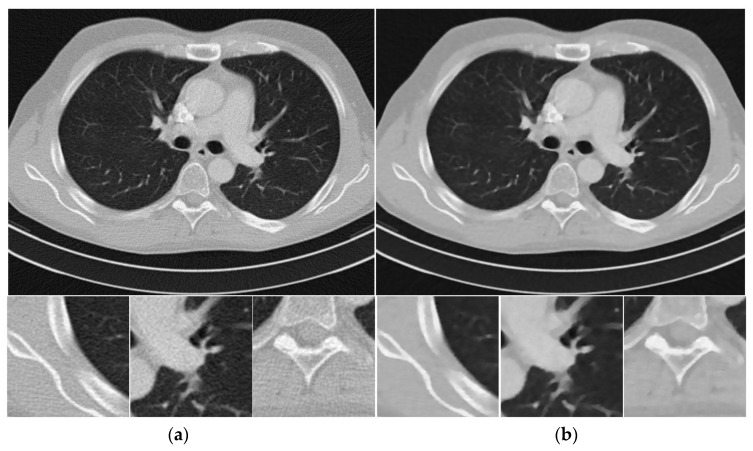

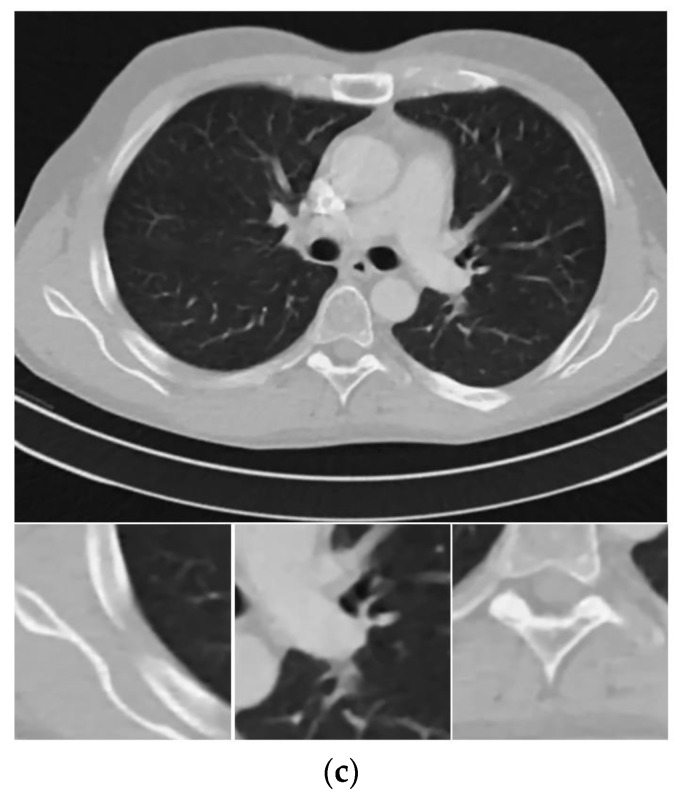
Thorax CT images reconstructed by (**a**) FBP, (**b**) SIR-TV, and (**c**) SIR-STV_1_ methods from noisy projections with 5 × 10^4^ incident photon number. The display window is [−1000, 667] HU.

Figure 4 shows the reconstructed images from noisy projections with incident photon numbers 5 × 10^4^ by the FBP, SIR-TV, and SIR- STV_1_ methods. In the proposed SIR-STV_1_ method,σ is set to 0.5, $L_{K}$ is set to be 3, and λ is set to $2\times 10^{-6}$. It can be seen that SIR-TV and SIR-STV_1_ can remove noise effectively and the SIR-STV_1_ method performs better in eliminating blocky effect than SIR-TV.

#### 3.2.2. Quantitative Comparison

To quantitatively evaluate the performance of the SIR-STV_1_ method, we compare the performance of the three methods on the reconstruction of ROIs with detailed structures, which were marked by red rectangles in Figure 1b. The quantitative results are given in Table 2. It can be seen that the SIR- STV_1_ method has the lowest RRE and highest SSIM for all of the ROIs. 

#### 3.2.3. Profile-Based Comparison 

**Figure 5:**
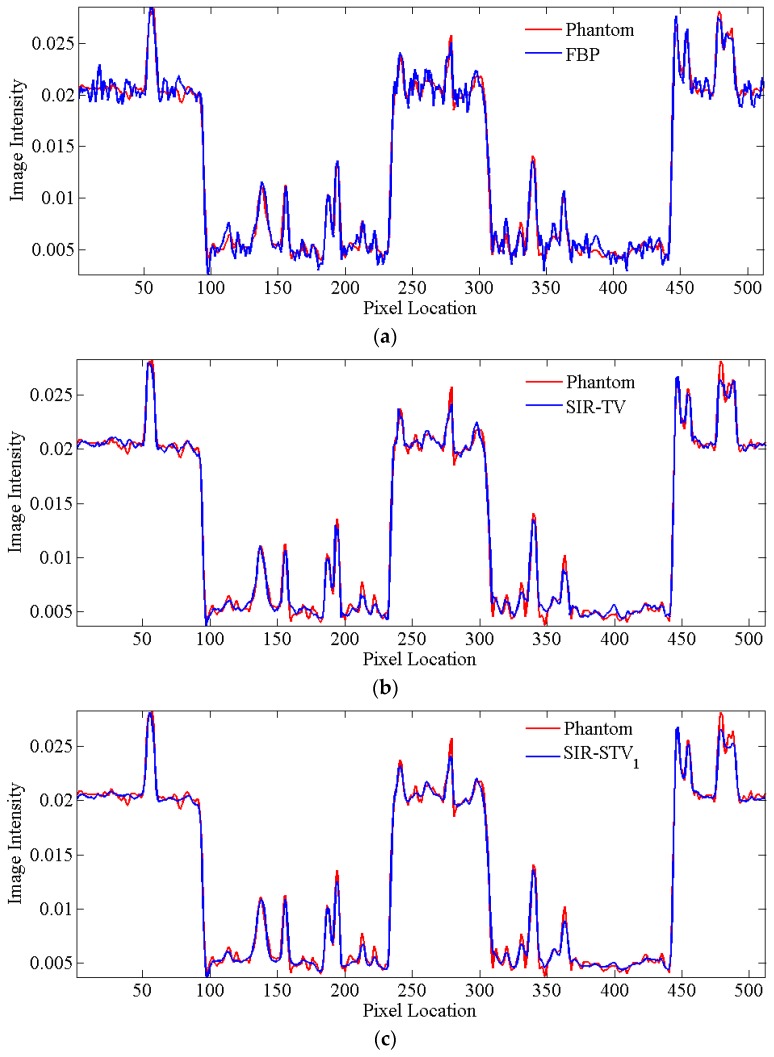
Comparison of the target profile in Figure 1b of different methods. (**a**) FBP; (**b**) SIR-TV; (**c**) SIR-STV_1_.

In order to further visualize the differences among the three methods in this Thorax numerical simulation experiment, the profiles of the resulting images corresponding to the white line in Figure 1b were plotted in Figure 5. It can be demonstrated that the profiles of the Thorax images reconstructed by the SIR-STV_1_ method achieve the best performance in terms of noise suppression and fine structure preservation.

#### 3.2.4. Analysis of the Parameter

**Figure 6:**
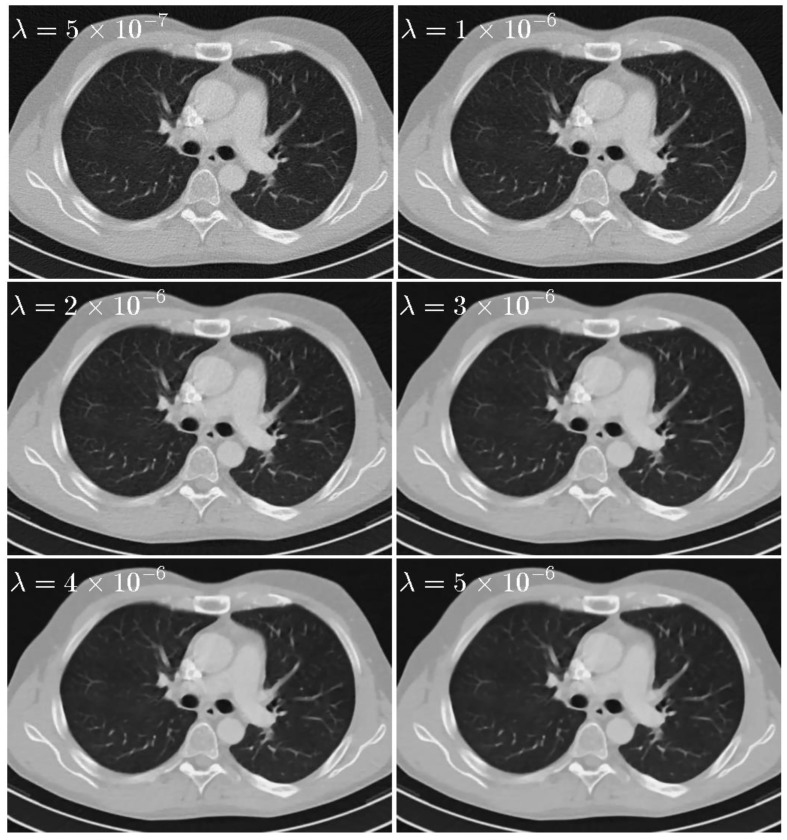
Thorax images reconstructed by the SIR-STV_1_ method with respect to different λ. The display window is [−1000, 667] HU.

**Figure 7:**
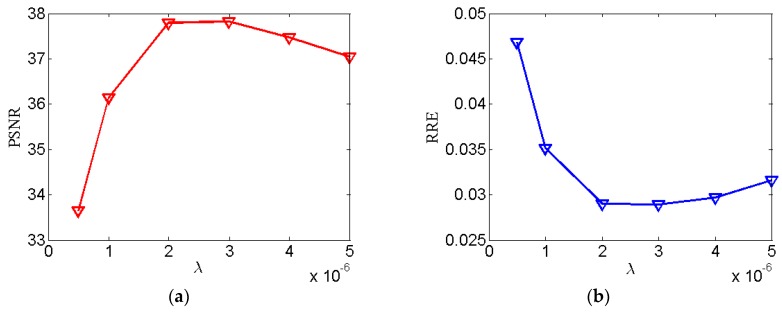

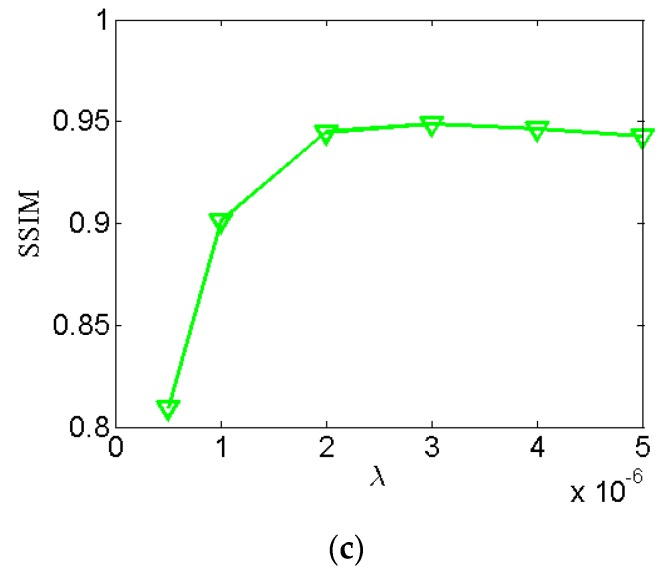
Image quality assessments of reconstructed Thorax phantom images for the SIR-STV_1_ method with respect to different λ. (**a**) The PSNR curve; (**b**) The RRE curve; (**c**) The SSIM curve.

(1) Regularization parameter λ: To sense the sensitivity of λ, experiments are performed with various $\lambda =5\times 10^{-7}$, $1\times 10^{-6}$, $2\times 10^{-6}$, $3\times 10^{-6}$, $4\times 10^{-6}$, and $5\times 10^{-6}$ with σ=0.5, $L_{K}=3$. The reconstruction images are shown in Figure 6. The PSNR, RRE, and SSIM curves corresponding to different λ are plotted in Figure 7. It is clear that the value of PSNR and SSIM are highest and the value of RRE is lowest when $\lambda =2\times 10^{-6}$.

(2) Parameters σ and *L_K_*

**Figure 8:**
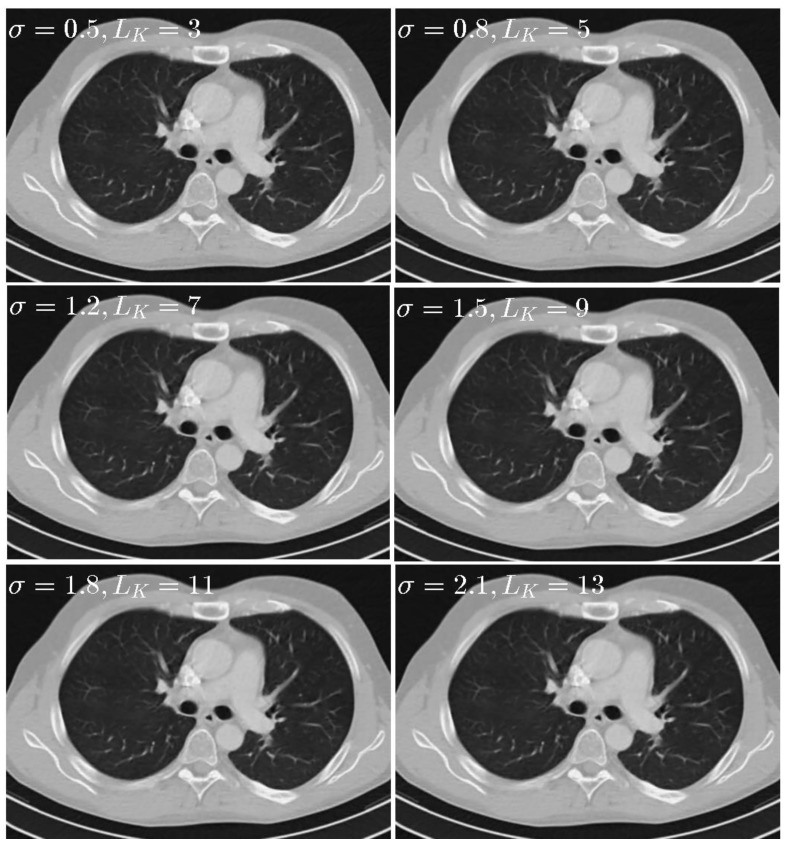
Thorax images reconstructed by the SIR-STV_1_ method with respect to different *σ* and *L*_*K*_. The display window is [−1000, 667] HU.

When σ was set to 0.5, Gaussian kernel with 99.7% of its energy will be within three pixels, so *L*_*K*_ and *σ* should be changed together instead of separately. To sense the impact of parameter σ and *L*_*K*_, six different sets of parameters including $\sigma =0.5,L_{K}=3$, $\sigma =0.8,L_{K}=5$, $\sigma =1.2,L_{K}=7$, σ=1.5, $L_{K}=9$ σ=1.8, $L_{K}=11$, and $\sigma =2.1,L_{K}=13$ are tested. The results are given in Figure 8. In addition, the quantitative results are given in Table 3. The results show that the performance of our proposed method is not quite sensitive to parameter *L*_*K*_ and *σ* and that there are no noticeable difference for different parameters. To balance the performance and computational time, the σ is set to 0.5, *L*_*K*_ is set to 3 in this paper.

**Figure 9:**
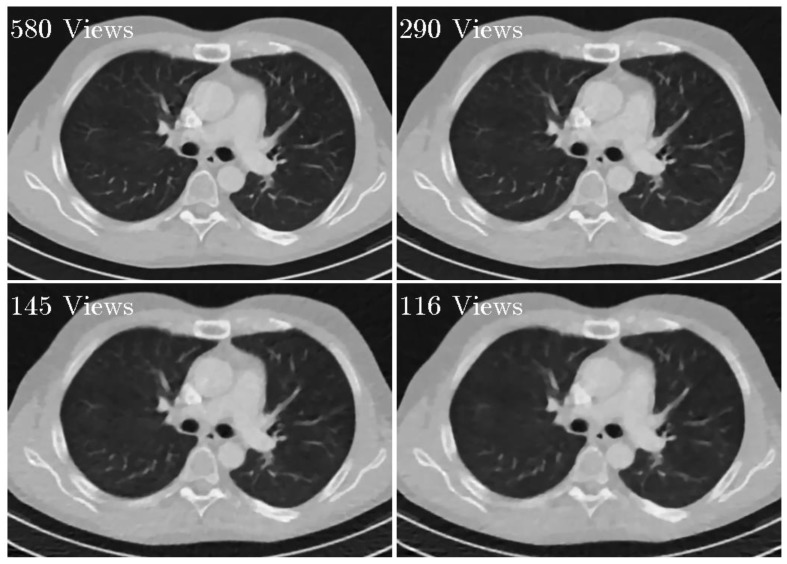
Reconstructed images using the SIR-STV_1_ method with respect to different views. The display window is [−1000, 667] HU.

(3) Number of projections: 

To evaluate the impact of the number of projections, simulated experiments with 580, 290, 145, and 116 views were performed. For all experiments, σ were set to 0.5, $L_{K}$ were set to be 3. When the number of projection view decreases, the regularization parameter λ should be increased accordingly to obtain high quality reconstructed results. For the reconstructions from 580, 290, 145, and 116 views, λ were set to 3 × 10^−6^, 4 × 10^−6^, 6 × 10^−6^, and 8 × 10^−6^, the number of subset were set to 20, 10, 5, and 4, respectively. The reconstruction results are shown in Figure 9. It can be observed that our proposed method can suppress the noise effectively. In the case of 580 views, the reconstruction result was almost as good as that reconstructed from 1160 views in Figure 4c. In the cases of 290, 145, and 116 views, our proposed method still obtains high quality reconstruction results. 

### 3.3. Realistic Sheep Lung Experiments

To validate the effectiveness of our proposed method for real data, an anesthetized sheep lung was scanned at normal and low dose, respectively on a SIEMENS Somatom Sensation 64-Slice CT Scanner in a circular cone-beam scanning mode. A scan protocol was developed for low dose studies with ECG gating: Time point 1 for a normal X-ray dose scan (100 kV/150 mAs) before a contrast agent injection, and time points 2–21 for low dose scans (80 kV/17 mAs) after the contrast agent injection. All the sinograms of the central slice were extracted, which were in a fan-beam geometry. The radius of the trajectory was 57 cm. A total of 1160 projections were uniformly collected over a 360° range. For each projection, 672 detector elements were equi-angularly distributed to define a FOV of 25.05 cm in radius. In this experiment, the reconstructed images were 768 × 768 pixels with a physical size of 50 × 50 cm. To further demonstrate the performance of our proposed method for a few views of CT reconstruction, 580 and 290 views projection were uniformly exacted form 1160 views projection. In our proposed algorithm, σ=0.5, $L_{K}$ = 3. For the reconstruction from noisy projections with 1160, 580, and 290 views, the parameter λ were set as 5 × 10^−6^, 7 × 10^−6^, and 2 × 10^−5^, the number of subset were set to 40, 20, and 10, respectively.

**Figure 10:**
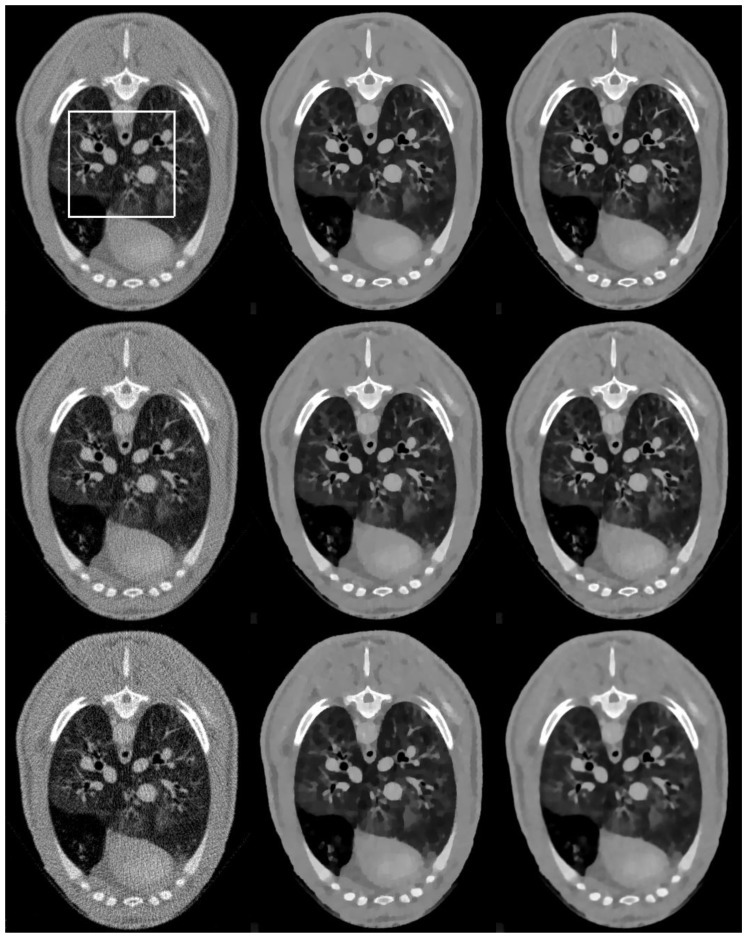
Images reconstructed by the FBP (left column), SIR-TV (middle column), and SIR-STV_1_ (right column) methods from low dose projections with 1160 views (the first row), 580 views (the second row, and 290 views (the third row). The display window is [−1000, 800] HU.

**Figure 11:**
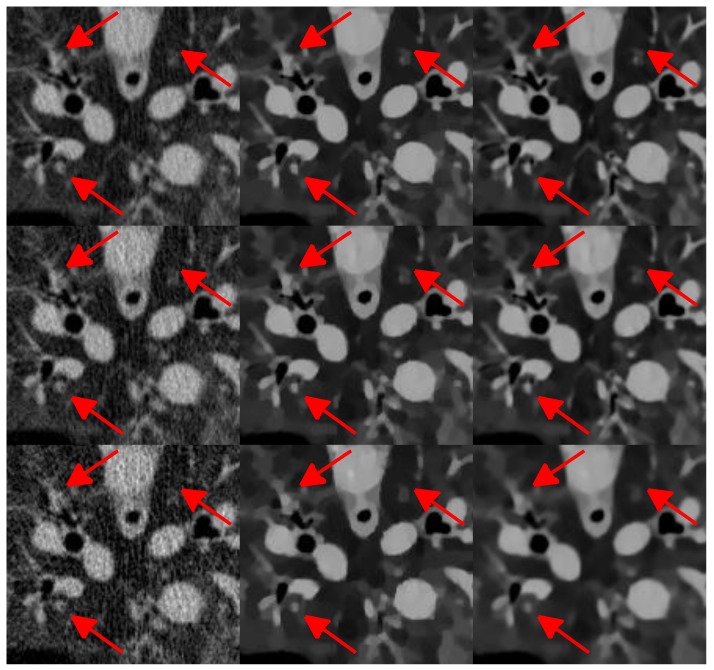
Zoom in view ofregion of interest(ROI) in Figure 10.

The reconstruction results are presented in Figure 10. It can be seen that the FBP results look very noisy and became worse and worse when the number of projection views decreased from 1160 to 290. Both the SIR-TV and our SIR-STV_1_ method outperform the FBP algorithm in suppressing image noise. To clearly compare the reconstruction performance of all algorithms, ROIs are extracted from Figure 10 and magnified in Figure 11. From Figure 11, especially denoted by red arrow regions, we can observe that the SIR-TV method produces patchy artifacts obviously, while the SIR-STV_1_ method could avoid the patchy artifacts effectively.

## 4. Discussion and Conclusions

With the development of STV_1_ in recent years, STV_1_ has been applied in many medical imaging problems, including multi-energy CT, dynamic myocardial perfusion CT, etc. Instead of penalizing the image at every pixel, STV_1_ considers the available information from the neighborhood of every pixel by penalizing the eigenvalue of structure tensor at this point. In this paper, we introduced the STV_1_ penalty into the SIR reconstruction framework for low dose CT reconstruction. Comparison studies among FBP and the SIR-TV method revealed that the proposed STV_1_ penalty effectively removed image noise and restrained the staircase effect of the TV penalty.

There are three parameters, the standard deviation of Gaussian convolution kernel σ, the radius of Gaussian convolution kernel *L*_*K*_, and the regularization parameter λ that needs to be adjusted. Extensive experiments in Section 3.2.4 reveal that the performance of our proposed method is not quite sensitive to the parameters *L*_*K*_ and *σ*. Therefore, to balance the reconstruction performance and computational complexity, we chose *σ* = 0.5 and *L*_*K*_ = 3 in our paper. In addition, from our experience this choice is also suitable for a wide range of applications. For the selection of λ, a large number of λ was manually tried to yield an optimal one in simulated experiments and realistic sheep lung data studies on the condition with fixed *L*_*K*_ and *σ*. It is known that this will require a large computation time and cost. In the future, we would study a regularization parameter selection method to obtain an approximate optimal regularization parameter for the presented AFISTA.

The OS-SQS method is massively parallelizable and well suited to modern computing architecture such as graphics processing unit (GPU), which is applied to replace the gradient descent method for minimizing the data fitting term in the original FISTA. Experimental results in Section 3.1.1 have demonstrated that the proposed AFISTA method converges much faster than FISTA. However, the convergence behavior of the AFISTA may be affected by the number and ordering of subsets, that is the proposed algorithms in some cases become unstable when it is too large. In the future, we would study how to balance the number of subsets and the convergence of the AFISTA. Meanwhile, stochastic gradient methods are investigated to realize the random ordering of the subsets.

Another important issue is the computation time. MATLAB codes are developed on a PC with a 3.20 GHz Intel core i7 processor and a 16 GB RAM. Our proposed AFISTA algorithm includes an image updating step and STV_1_-based denoising step. In brain image numerical simulation with 1160 views, the image updating step and STV_1_-based denoising step in each iteration takes 2.3463 s (on average) and 40 s (on average), respectively. GPU was employed in the projection and back-projection operation to speed up the proposed algorithm. In order to further improve the reconstruction speed of the AFISTA, we would use GPU in STV_1_-based denoising step.

In conclusion, we introduced the STV_1_ regularizer into SIR framework for low dose CT reconstruction and developed an effective algorithm to minimize the objective function using AFISTA. Both simulated data and realistic sheep data were used to validate the proposed method. From the experiments, we have seen that the proposed SIR-STV_1_ have better performance in restraining the stair effect and noise suppression than FBP and SIR-TV method. Additionally, in the future, the proposed SIR-STV_1_ framework will be refined and adapted to handle other topics in CT imaging, such as few-view reconstruction and interior CT.

## Figures and Tables

**Table 1:** Image quality metrics.

Incident Photon Number	Algorithm	PSNR (dB)	RRE	SSIM
5 × 10^3^	FBP	26.6131	0.2049	0.5526
	SIR-TV	33.8416	0.0892	0.7539
	SIR-STV_1_	35.7597	0.0715	0.7639
1 × 10^4^	FBP	29.8176	0.1417	0.6135
	SIR-TV	35.7454	0.0716	0.8162
	SIR-STV_1_	38.2683	0.0536	0.8248
5 × 10^4^	FBP	36.7103	0.0641	0.7318
	SIR-TV	40.2409	0.0427	0.9016
	SIR-STV_1_	42.8079	0.0307	0.9275

**Table 2:** A summary of the evaluation indexes of the reconstructed image at different noise levels in the Thorax image simulated studies.

Different ROI	Algorithm	PSNR (dB)	RRE	SSIM
ROI 1	FBP	30.7537	0. 0447	0.7403
	SIR-TV	35.0948	0.0271	0.9206
	SIR-STV_1_	35.7648	0.0251	0.9370
ROI 2	FBP	29.9316	0.0540	0.7730
	SIR-TV	33.1043	0.0374	0.9145
	SIR-STV_1_	34.2960	0.0326	0.9348
ROI 3	FBP	30.0721	0.0446	0.7021
	SIR-TV	33.8564	0.0288	0.8927
	SIR-STV_1_	34.9798	0.0253	0.9183

**Table 3:** A summary of the evaluation indexes of the reconstructed image in Figure 8.

Parameter	PSNR (dB)	RRE	SSIM
σ=0.5, *L*_*K*_ = 3	37.7983	0.0290	0.9449
σ=0.8, *L*_*K*_ = 5	37.9762	0.0283	0.9477
σ=1.2, *L*_*K*_ = 7	37.9653	0.0284	0.9482
σ=1.5, *L*_*K*_ = 9	37.8765	0.0287	0.9480
σ=1.8, *L*_*K*_ = 11	37.7748	0.0291	0.9474
σ=2.1, *L*_*K*_ = 13	37.6704	0.0294	0.9466
